# Predicting reference soil groups using legacy data: A data pruning and Random Forest approach for tropical environment (Dano catchment, Burkina Faso)

**DOI:** 10.1038/s41598-018-28244-w

**Published:** 2018-07-02

**Authors:** Kpade O. L. Hounkpatin, Karsten Schmidt, Felix Stumpf, Gerald Forkuor, Thorsten Behrens, Thomas Scholten, Wulf Amelung, Gerhard Welp

**Affiliations:** 1 University of Bonn, Institute of Crop Science and Resource Conservation (INRES), Soil Science and Soil Ecology, Nussallee 13, D-53115 Bonn, Germany; 2 University of Tübingen, Department of Geosciences, Soil Science and Geomorphology, D-72070 Tübingen, Germany; 3 Agroscope – Swiss Federal Research Institute for Sustainability Soil Monitoring Network (NABO) – Modelling Unit, Reckenholzstrasse 191, CH - 8046 Zürich, Switzerland; 4 West African Science Service Centre on Climate Change and Adapted Land Use – WASCAL, Ouagadougou 06, P.O. Box 9507, Burkina Faso

## Abstract

Predicting taxonomic classes can be challenging with dataset subject to substantial irregularities due to the involvement of many surveyors. A data pruning approach was used in the present study to reduce such source errors by exploring whether different data pruning methods, which result in different subsets of a major reference soil groups (RSG) – the Plinthosols – would lead to an increase in prediction accuracy of the minor soil groups by using Random Forest (RF). This method was compared to the random oversampling approach. Four datasets were used, including the entire dataset and the pruned dataset, which consisted of 80% and 90% respectively, and standard deviation core range of the Plinthosols data while cutting off all data points belonging to the outer range. The best prediction was achieved when RF was used with recursive feature elimination along with the non-oversampled 90% core range dataset. This model provided a substantial agreement to observation, with a kappa value of 0.57 along with 7% to 35% increase in prediction accuracy for smaller RSG. The reference soil groups in the Dano catchment appeared to be mainly influenced by the wetness index, a proxy for soil moisture distribution.

## Introduction

Soils play a vital role for various ecosystem services, which makes them a key asset for sustainable living conditions on earth. Major soil functions are related to food and biomass production, water control, biological and chemical recycling, platform provision for human activities, supply of raw materials, and providing habitat for soil biodiversity^1^. However, many countries still need to deal with the lack of adequate and timely soil information to address e.g. land degradation issues. This is especially true in sub-Saharan Africa, where logistical challenges result in a severe limitation in data availability and storage^2^. Additionally, traditional soil mapping is known to be very expensive and time-consuming. As early as 2005, it was reported that most of the soil information maps in many sub-Saharan countries had been lost^3^.

In many African countries, the low quality of existing soil information can be related to poor georeferencing records, irregularities between laboratory and mapping approaches, inconsistencies in legends and field survey reports^4^, and different levels of landscape perception and mapping experience^5^. Moreover, existing soil maps are mostly produced at a coarse scale and are derived from traditional qualitative surveys^6^. Dewitte *et al*.^7^ observed that most African countries still depend on the 1:5 M “Soil Map of the World” produced by FAO and UNESCO in the 1970s. These maps usually cover the whole country, but are inadequate for local applications due to insufficient resolution of the data^8,9^. Initiatives such as the GlobalSoilMap.net project are currently working to overcome the previous challenges in order to provide up-to-date and relevant soil information in Africa using present-day techniques^10^. However, most soil maps have focused primarily on soil properties, while soil class maps are equally important for allocating a particular soil unit for a specific use^11^. Moreover, the improvement of soil resilience requires that management approaches tailored to the specific characteristics of the targeted soil classes^12^.

As a time- and cost-effective alternative to classical soil surveys, digital soil mapping (DSM)^13^ is a subset of pedometrical research that uses geo-statistics and data mining methods to spatially predict soil classes or soil properties based on existing soil and environmental covariate data. These relations are described by the “scorpan” function developed by McBractney *et al*.^13^ and is based on the soil state-factor equation initiated by Jenny^14^. The “scorpan” function introduces a conceptual framework for quantitative pedology and is defined as follows:

1 $${S}_{c}=f(s,\,c,\,o,r,p,a,n)$$

where *Sc* is soil class, *s* is for soils and other soil attributes (mainly relates to pedotransfer functions), *c* is climate, *o* is organism (vegetation, fauna, or human activity), *r* is relief (topography), *p* is parent material (lithology), *a* is age, *n* is spatial location, and $f$ is function or soil spatial prediction function (SSPF) model.

The SSPF represents a wide range of statistical and mathematical methods used in DSM for estimating soil classes at undocumented areas with similar environmental conditions. Models used for soil taxonomic class prediction include linear discriminant analysis^15^, logistic regression^16^, support vector machines (SVM)^17^, artificial neural networks (ANN)^18^, and decision trees (DTs)^19–21^. The latter have become more popular in DSM and among the most commonly used DTs algorithms are C4.5/SEE5^22^, CART (Classification and Regression Trees)^23^, and Random Forest (RF)^24–26^.

DTs use a hierarchical top-down approach that recursively partitions the covariate space using a binary split resulting in branch-like divisions^13^. RF, an extension of DTs, has gained tremendous popularity in DSM in the last decade. RF can be regarded as a non-parametric ensemble learner that cumulates a set of highly randomized classification trees to provide prediction^27^. The RF algorithm uses repeatedly drawn sample sets with replacement (bootstrap sampling) with the same size as the training set for the construction of the individual trees. Additionally, a random subset of the covariate space is used to split a node during the construction of each tree^27,28^. The RF algorithm is advantageous because of its ability to handle numerical and categorical data without any assumption of probability distribution, robustness against nonlinearity and overfitting^29^. Moreover, the RF provides measures of the most important covariates involved in model accuracy^30,31^. The application of RF has been reported for the classification of crop types^32^, tree species^33^, soil parent material^29^, as well as soil taxonomic units^34–36^.

In the present study, the RF was used for soil classification. Though other machine learning algorithms could be used or added might warrant further attention for different geographic settings, previous studies that compared many machines learning algorithms pointed out that differences in prediction accuracies were often marginal with the RF providing in most cases a higher overall accuracy^37–39^. A similar level of accuracy was observed between RF and SVM by Adam *et al*.^37^ and between RF and stochastic gradient boosting (SGB) by Freeman *et al*.^38^ Inglada *et al*.^40^ noted a better performance in crop mapping for RF as compared to SVM, SGB and decision trees. The same conclusion was reached by Rodriguez-Galiano *et al*.^41^ when comparing SVM, ANN and regression trees for complex land cover and land use classes. Brungard *et al*.^39^ compared 11 machine learning algorithms including SVM and ANN for soil classification and reported that RF provided the most consistent accuracy in soil classification. Heung *et al*.^17^ tested the performance of a set of machine learning methods – for example CART, k-nearest neighbor, nearest shrunken centroid, ANN, multinomial logistic regression, logistic model trees, SVM and RF – to segregate between a complex set of soil orders and suborders. They reported RF among the preferred due to its fast parameterization, high accuracy and the interpretability of the output.

The environmental covariates used in DSM are mostly terrain attributes, since these data are generally available for study areas. Remote sensing imagery data (spectral data) were also considered along with terrain attributes in some studies^42,43^. The present study focused on covariates derived from terrain attributes from the Shuttle Radar Topography Mission (SRTM) digital elevation model (DEM) with a 90 m resolution^44^, multi-temporal RapidEye and Landsat imagery bands, and indices calculated from them, along with maps of parent material, climate and land cover data, to unravel the complex soil environmental relations regarding spatial soil class distribution. However, in order to reduce the complexity of the covariate space and optimize prediction accuracy, a feature selection approach was used to exclude irrelevant and/or redundant information^39,45^. Recursive feature elimination has been proven to objectively reduce high-dimensional data sets in machine learning algorithms, while preserving the prediction accuracies^39,46,47^.

The soil data used together with covariates in DSM are mostly legacy data encompassing soil classes and/or soil properties^48,49^. However, for collecting legacy data, surveyors with different landscape perceptions and field experiences are usually involved. As a result, there is a potential risk that a bias may be introduced when discriminating between soil classes in the field^50,51^. These errors are mostly formulated as source errors^52^.

Ideally, the quantitative relationship of soil classes with covariates is expected to be distinct enough to differentiate between classes. However, in complex landscapes, the sharing of similar landscape positions by soil classes could result in overlapping covariate space, with the most abundant class dominating the overall classification^53^. Balanced datasets are ideal to allow decision trees algorithms to produce better classification^54^. Generally, no statistical design is taken into consideration for traditional soil sampling resulting in the legacy soil data which are used for DSM^55^, especially in some data-scarce countries in Africa. For datasets with uneven class size, the generated classification model generally biases towards the majority class^54^. Many studies have been conducted along these lines, but most have focused on binary classes^56,57^, while the current paper uses a multiple set of soil groups. The present study deals with a large number of soil samples (n = 987) related to six reference soil groups with Plinthosols being the dominant group. The Plinthosols were found to share the similar landscape position to a certain extend with minor soil classes that therefore could be misclassified by the different involved surveyors, or which could merely be underrepresented using random sampling within conventional DSM mapping approaches.

Strategies proposed for dealing with imbalanced dataset range mainly from affecting specific costs to training set^58,59^, re-sampling the training set, either by over-sampling the minority class^60,61^, and/or under-sampling the majority class^62,63^. Many variants of these techniques exist and have been reviewed by López *et al*.^64^ and Prati *et al*.^65^. In our context, the aim was to reduce the potential misclassification within the training set by (1) using an objective reduction technique, based on identifying important predictors, (2) using these predictors to objectively reduce the training set number, and (3) predicting the classes with an overall higher accuracy. We used an undersampling approach by reducing the sample size of the Plinthosols in order to improve the prediction accuracy of the smaller soil groups. Taking the imbalance distribution into account, we pruned the majority class by removing samples belonging to the outer ranges of the most important covariate driving the overall RF classification. We examined whether data subsets generated by the pruning of the Plinthosols would result in an increase of prediction accuracies by using RF as a robust method to evaluate the various sets. Although imbalanced data has been used in many studies dealing with soil classification^34,39,66^ no such method has, to our knowledge, been applied for legacy soil data from a tropical semi-arid environment. In addition, we compared this method with the random oversampling (ROS) approach^59,60^. Having considered the pruning approach, we hypothesized that instance selection on the majority soil group, along with model-based feature selection, would improve the performance of the RF models and result in a stronger response of the minority soil groups.

## Materials and Methods

### Study area

**Figure 1:**
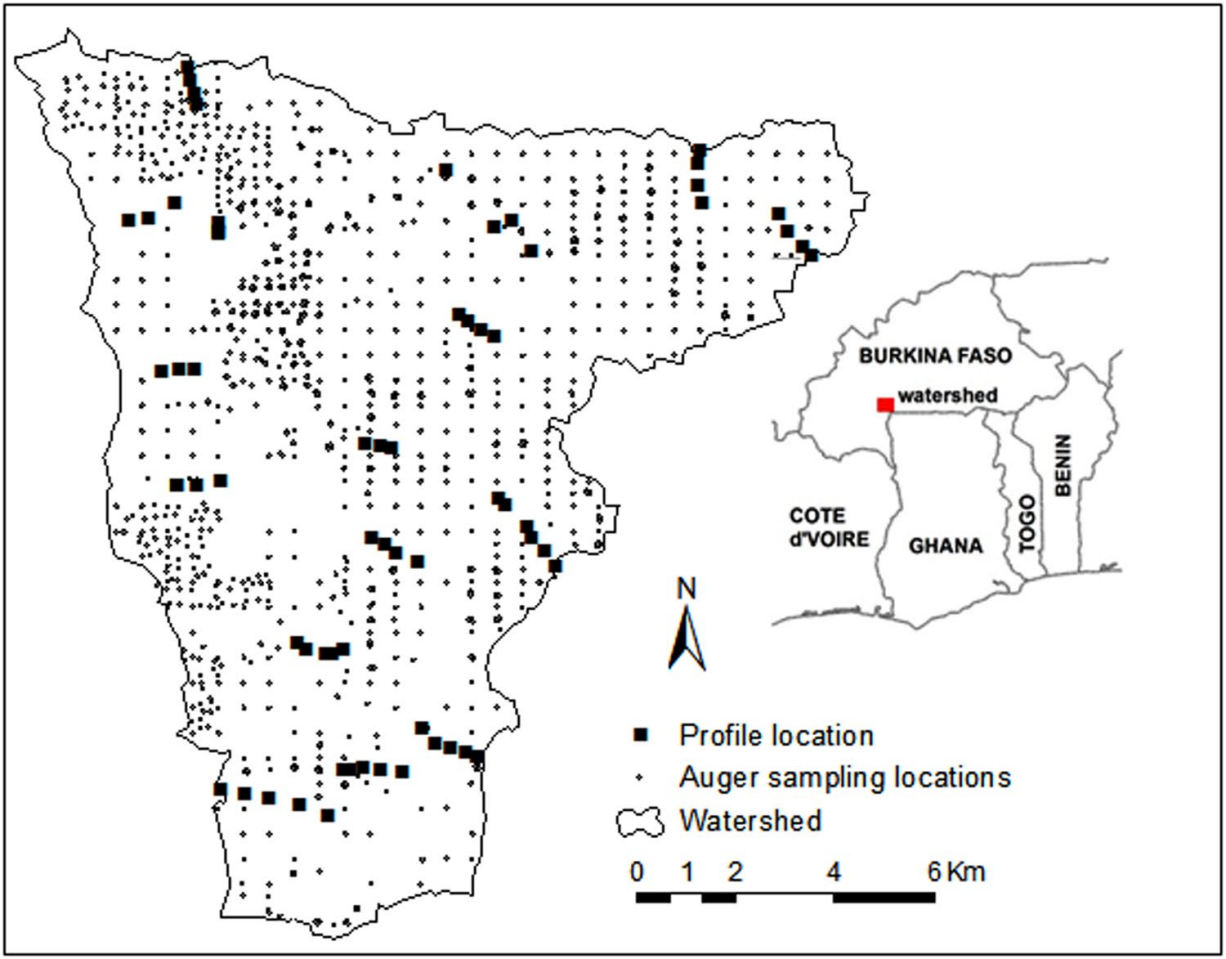
Map of the study area with profile and auger sampling locations (the map was generated using ESRI ArcMap 10.3.1, www.esri.com).

The study area – the Dano catchment – is located in the Ioba province, in the southwest of Burkina Faso (Fig. 1). The area comprises approximately 155 km^2^ and its altitude ranges from 259 to 465 m asl, with an average slope gradient of 3.6%. The mean annual temperature is approximately 28 °C and the annual precipitation ranges from 800 mm to 1200 mm, with only one rainy season generally covering the period of May to October. The lithology is characterized by precambrian metavolcanites, which are mainly andesic rocks^67^.

### Soil Sampling and reference soil groups

The dataset used in the present study was collected in different campaigns and involved many surveyors. Firstly, 70 soil profiles were described by different surveyors along 16 transects from August to October 2012 (Fig. 1). The soil profile locations were chosen following a toposequence covering different reference soil groups and land uses. These profiles were excavated up to 1 m. Moreover, an intensive auger grid sampling was carried out over the entire study area in 2012 and 2013. The sampling campaigns were conducted by the surveyors from August to October for both years and resulted in a total of 917 soil samples. At each sampled location, a detailed soil horizon description was carried out and soil classification was conducted according to the World Reference Base (WRB) for soil resources^68^.

Six soil classes were encountered in the Dano catchment and were described based on the WRB as follows: Cambisols, Gleysols, Lixisols, Leptosols, Plinthosols, and Stagnosols. The Cambisols are young soils with incipient soil formation with beginning horizon differentiation demonstrated by changes in color, structure, or carbonate content. Gleysols refer to water-influenced soils that are saturated with groundwater long enough to develop a characteristic “gleyic color pattern” made up of reddish, brownish, or yellowish colors at ped surfaces and/or in the upper soil layer(s), along with greyish/bluish colors inside the peds and/or deeper in the soil. Stagnosols are also water-influenced soils that are characterized by a perched water table showing redox processes caused by surface water due to periodical wetting; they are mottled in the topsoil and subsoil, with or without concretions and/or bleaching. Lixisols consist of strongly weathered soils in which clay has been removed from an eluvial horizon down to an argic subsurface horizon that has low-activity clays and a moderate-to-high base saturation level. Leptosols include very shallow soils over hard rock or very calcareous material, but also deeper soils that are extremely gravelly and/or stony. Plinthosols point to soils that contain “plinthite”; that is, a humus-poor mixture of Fe oxides and kaolinitic clay with quartz and other materials that change irreversibly to a hardpan or to irregular aggregates on exposure to repeated wetting and drying. For more detailed description, refer to IUSS *et al*.^68^.

### Geospatial and spectral variables

To provide a wide range of different environmental covariates dealing with the state factor equation, we compiled a set of predictors (Tables 1 and 2), from different sources using ArcGIS 10.3.1 (Environmental Systems Research Institute, ESRI Inc., Redlands, CA) and SAGA GIS (System for Automated Geoscientific Analyses). The covariates used in this study were terrain attributes (Table 1), spectral bands and indices (Table 2) and climate variable (precipitation). The terrain attributes were derived from a SRTM (Shuttle Radar Topography Mission) DEM with a 90 m resolution^69^. As climatic information we used annual precipitation (Prep) at 1 km resolution from the WorldClim datasets^70^.

**Table 1:** Terrain attributes used as predictors for soil mapping. *Variables computed with both ArcGIS and SAGA.

Variables	Abbreviation	Unit
Distance to stream ArcGis	Dist.stream	m
Elevation ArcGis	Elevation	m
Flow direction ArcGis/SAGA	A.Flow.d/S.Flow.d*	—
Flow accumulation ArcGis/SAGA	A.Flow.A/S.Flow.A	—
Plan curvature ArcGis	A.Plan.curv/S.Plan.curv	degree m^−1^
Total curvature SAGA	S.totalcuv	degree m^−1^
Flow line curvature SAGA	S.Flow.line.curv	degree m^−1^
Catchment Area Parallel SAGA	S.CA.Par	m^2^
Aspect ArcGis	A.Asp	—
Northness	cose.Asp	Degree
Topographic Wetness Index ArcGis/SAGA	A.TWI/S.TWI	—
SAGA Wetness Index SAGA	S.Wet.Ind	—
Terrain ruggedness SAGA	Terr.Rugg	
Protection index SAGA	Prot.Index	—
Overland flow distance SAGA	Overland.Flow.dist	m
Horizontal flow distance SAGA	S.HF.dist	m

**Table 2:** Spectral bands and soil/vegetation indices derived from the RapidEye and Landsat data.

Source	Total number of Bands	Band number, names and abbreviations					
		1	2	3	4	5	6
RapidEye	5	Blue (B)	Green (G)	Red (R)	Red edge (RdE)	Near infra red (NIR)	—
Landsat	6	Blue (B)	Green (G)	Red (R)	Near infra red (NIR)	Shortwave infrared 1 (SWIR 1)	Shortwave infrared 2 (SWIR 2)
Computed Spectral indices							
Name of Index		Formula			Index description		
Brightness Index (BI)		${(({R}^{2}+{G}^{2}+{B}^{2})/3)}^{0.5}$			Average reflectance magnitude		
Saturation Index (SI)		$(R-B)/(R+B)$			Spectral slope		
Hue Index (HI)		$(2\ast R-G-B)/(G-B)$			Primary colors		
Coloration Index (CI)		$(R-G)/(R+G)$			Soil color		
Redness Index (RI)		${R}^{2}/(B\ast {G}^{3})$			Hematite content		
Normalized Difference Vegetation Index		$(NIR-R)/(NIR+R)$			Health and amount of vegetation		

The spectral data (Table 2) were obtained from two optical sensors: RapidEye and Landsat 8. They were acquired on March 1, April 1, and May 3, 2013 (RapidEye) and June 13, 2013 (Landsat). These periods correspond to the peak of the dry season and the tilling period in which soils are mostly bare, especially in farming areas. The RapidEye Science Archive team of the German Aerospace Center (DLR) (https://resa.blackbridge.com/) provided the RapidEye data, while the platform of the United States Geological Survey’s GLOVIS website (http://glovis.usgs.gov) was used to obtain the Landsat 8 data. The RapidEye data were characterized by five spectral channels (blue, green, red, red-edge and near-infrared (NIR)) with a spatial resolution of 5 m. The Landsat data had 11 spectral channels^71^ with a 30 m spatial resolution, but only six channels (blue, green, red, NIR, shortwave infrared (SWIR1, SWIR2)) were used in the current study. An atmospheric correction of these images was carried out using the ENVI ATCOR software^72^. Specific soil indices^73^ were also computed in addition to the spectral bands (Table 2). Apart from the terrain attributes, resampling was done to transfer all datasets to the same spatial resolution of 90 m.

### Modeling with Random Forest and variable selection

The RF method is an ensemble learning technique that uses many classification trees to give predictions for the response variable^27^. A description of the RF algorithm is provided below^27^:

1. Take a bootstrap sample (random sampling with replacement) of size N, where N is the number of samples in the original dataset, mostly resulting in a sample distribution of ~63% of unique and of ~37% of multiple repetitions of instances. This is repeatedly done for each individual tree.
2. Build each tree T_i_ from the bootstrap samples by carrying out the following operations iteratively: at each tree node, select the best split among a randomly chosen subset of m_try_ (instead of all) covariates and divide the response variable accordingly. The tree is then grown until the number of instances within each terminal node has fallen below the number of nodesize cases.
3. Aggregate the predictions of all trees ${\{{T}_{i}\}}_{1}^{n}$ by using majority votes for classification.

The error rate is estimated as follows:

1. For each tree, prediction performance is measured using instances not in the bootstrap sample (designated as “out-of-bag” data, or OOB data) considering the T_i_ built within the bootstrap set.
2. Aggregate all individual OOB predictions and compute the model error rate, which is the overall proportion of misclassification.

In addition, the RF algorithm allows the determination of the importance of each covariate by calculating the mean decrease accuracy. Therefore, two steps are necessary for each covariate: (i) randomly permute the values of each covariate within the OOB set, and (ii) measure how much the permutation reduces the accuracy of the model. For less important variables, the permutation should have little to no effect on model accuracy, while permuting important variables should reduce it significantly.

The RF requires to set the number of trees to be grown in the forest (ntree) as well as the number of variables at each split (m_try_)^27^. For the current study, 500 trees were built and the number of features at each split was defined based on a grid-learning method implemented in the Classification and Regression Training (Caret) package in the R software^74^.

We tested the potential of the Recursive feature elimination based on RF^75^ to select an optimal set of covariates, from all variables, for classification. Recursive feature elimination works by establishing a classification model using all available covariates, then proceeds to rank these by order of importance, and discards the covariates of the lowest importance. It replicates the same process until all the covariates are eliminated^39^. The Recursive feature elimination was performed on the training set obtained after split of the dataset (see section Model validation and map comparison for more details). For the recursive feature elimination, a 10 fold cross-validation with 5 repetitions was carried out using the training set with the Caret Package^74^. The Recursive feature elimination algorithm requires the setting of statistical functions (linear models, decision trees, etc.), as well as an evaluation metric to select the optimal model. For that purpose, the RF was chosen as classifier while Kappa^76^ was used as the evaluation metric.

The optimal subset of covariates resulting from the Recursive feature elimination approach returned eight variables: wetness index, elevation, distance to stream to network, protection index, and precipitation, near infrared, and shortwave infrared. The RF modeling was then carried out using all covariates on the one hand, and the optimal set of covariates resulting from the recursive feature elimination (RF_rfe) on the other.

### Experimental design: Data pruning treatments

The field observation of this study revealed that the Plinthosols were the dominant reference soil group, with approximately 73% of the total number of samples (see Supplementary Table S1). These Plinthosols as described by the different surveyors were set in a similar landscape position along with minor soil classes resulting in potential misclassifications. To reduce the potential misclassification within the training set, an objective instance selection technique was conducted on the majority class – the Plinthosols. As a general assumption, we envisaged the possibility of a potential overestimation of this particular soil class, as is often the case for such large datasets with imbalance-related issues. Therefore, the first step in the present study was to test this hypothesis by running the model with the entire dataset. In a second step, data pruning was carried out as a method to address the potential prominence of the majority class in the covariate space once the latter hypothesis revealed true. For this purpose, a set of data pruning treatments was conducted by defining a set of data “core ranges” (CR). Throughout the paper, the term “core range” refers to the training data without the Plinthosols´ samples that fell into the outer range (as clarified below) of the most important covariate.

### Pruning treatment: Choosing major variable

The different pruning operations were carried out based on the variable importance measurement expressed by the RF mean decrease in classification accuracy. The latter follows the rationale that when values of a variable at a particular node are randomly permuted, this variable is supposedly absent from the model. The difference in the classification accuracy before and after the permutation of the values of the predictor variable – that is, after considering and excluding this predictor variable – is used as a measure of variable importance^77^. These computations are conducted tree by tree until the whole random forest is constructed^78^. This results in the discrimination between essential and non-essential covariates. The most essential covariate is the one with the highest contribution to model accuracy and with the greatest impact in the covariate space, driving the overall classification. Consequently, we used the most important covariates to determine the data CR for the pruning operation of the Plinthosols.

### Pruning treatment: data core range

**Figure 2:**
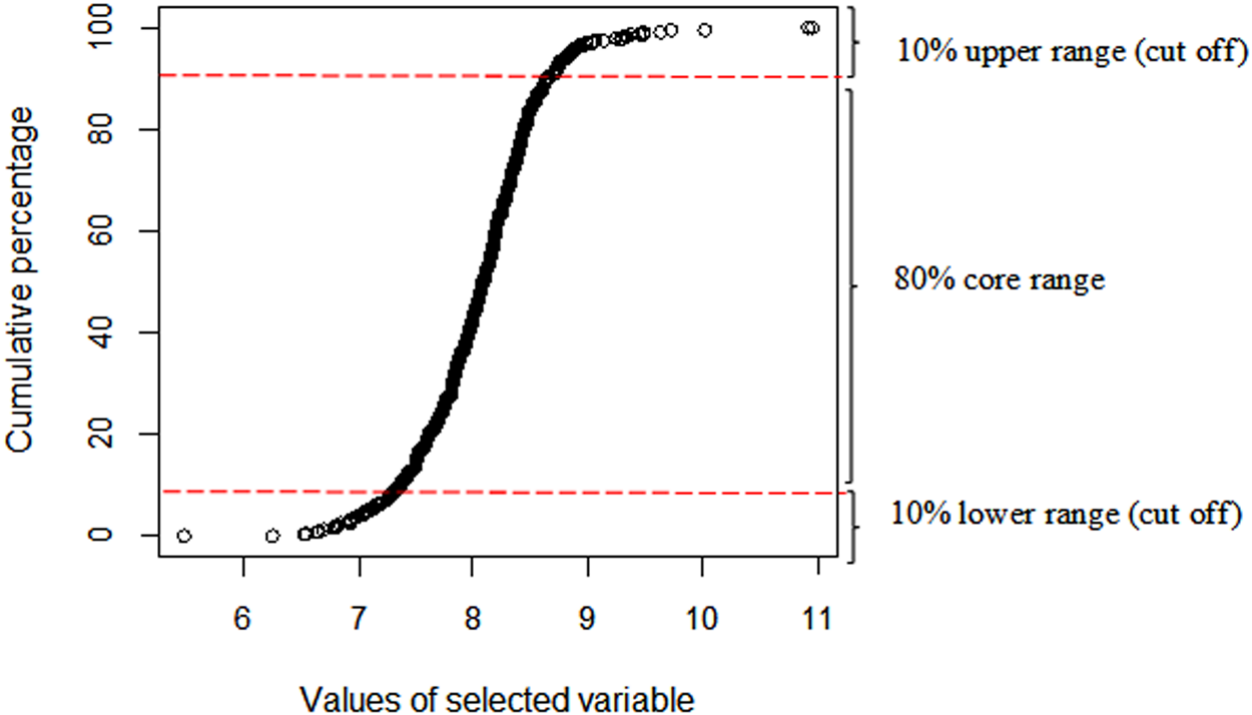
Core range data definition based on the cumulative percentage of the density distribution of the driving covariate and the cutting off of Plinthosol samples falling within the outer range (definition of 80% core range dataset).

**Figure 3:**
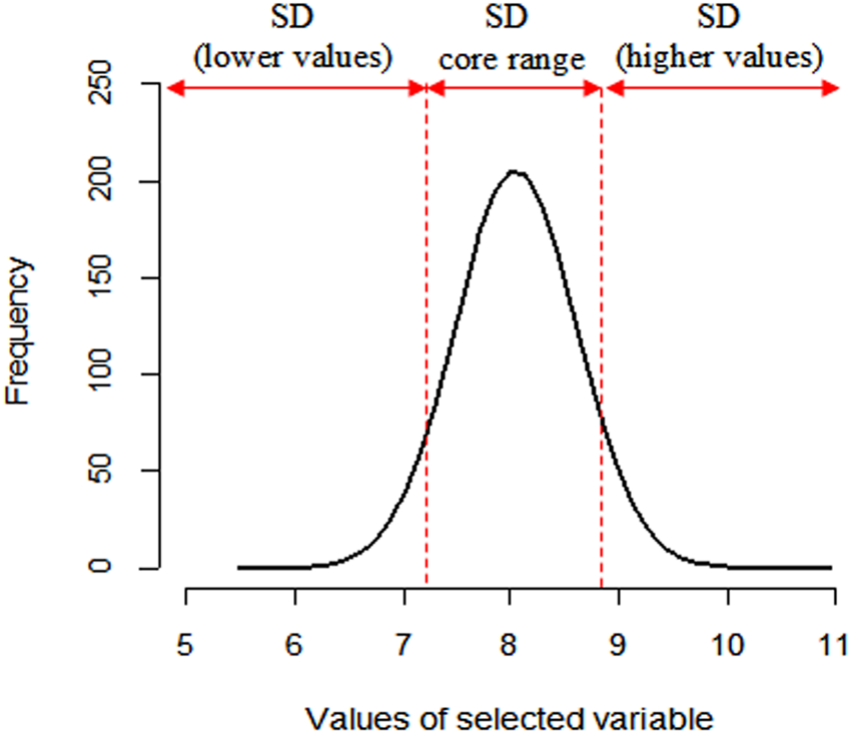
Core range data definition based on the standard deviation of the values of the driving covariate and the cutting off of Plinthosol samples falling within the outer range. SD: standard deviation.

The data pruning treatments were carried out by defining a set of 80% (80%CR) and 90% (90%CR) core range, as well as by a standard deviation (σ)-based (SDCR) core range while cutting off all data points of the Plinthosols belonging to the outer range of the most important covariates. The CR, as well as the remaining instances, were calculated as follows: (i) calculate the density distribution of the most important covariate, (ii) standardize the density distribution to a range of 0–100, and (iii) exclude all instances that fall into the margin; for example, for the 80%CR, we only used the instances within the interval of 10–80% (Fig. 2). Similarly, a CR based on the standard deviation (σ) of the values of the most important covariate was defined (Fig. 3). For that purpose, values lower than “μ − σ” (with μ being the arithmetic mean of the driving variable) were cut off, as were values higher than “μ + σ”. The standard-deviation-based core range (SDCR) was then set by considering data values within one standard deviation of the mean (mathematically, μ ± σ).

Four different datasets were defined: (1) entire dataset with all Plinthosols (AllPT), (2) a 90% core range (90%CR) of Plinthosol samples, (3) an 80% core range (80%CR) of Plinthosol samples by cutting off all points lower than 10% and higher than 90% of the cumulative percentage, and (4) a SD core range (SDCR) of Plinthosol samples by pruning values lower and higher than “μ − σ”and “μ + σ”, respectively. Each dataset was used to train a RF model using the terrain attributes and multispectral data.

### Random oversampling

The ROS approach was used to create a training dataset in which all the soil classes have an equal number of instances. ROS proceeds by randomly duplicating instances of the minority classes in order to match the number of samples in the majority class^79,80^. The ROS was conducted as carried out by Heung *et al*.^81^. Considering the 90%CR dataset for example, for 468 and 113 instances of the majority (Plinthosols) and minority (Gleysols) class respectively, ROS will draw 468 times with replacement of instances out of the 113 (see Supplementary Table S2). The “caret” R Package^74^ was used for the ROS of the training set of each of the defined dataset (AllPT, 90%CR, 80%CR, and SDCR). Modelling with each of the oversampled dataset was also carried out as conducted with the non-oversampled dataset.

### Model validation and map comparison

The dataset was split, with 80% used for training and 20% for validation. For consistency and stability of the different models, a 10-fold cross-validation approach with 5 repetitions was carried out for each dataset using the “caret” R Package^73^. The entire training set (AllPT) as well as the pruned datasets (80%CR, 90%CR and SDCR) were evaluated over the same validation data initially obtained as independent validation dataset. Since the OOB error is data specific, it is not a good measure for comparisons between different studies. Therefore, the classification accuracy of the different models was based on the Kappa index. The Kappa value (ϰ) gives the level of accuracy for a particular classification due to chance agreement^76^. This is particularly important when dealing with imbalanced class data, as a class with larger distribution would result in higher classification accuracy. We considered a ϰ value of 0 as a random classifier, 1 as perfect classification, 0.80 as strong agreement, between 0.4 and 0.8 as substantial agreement and below 0.4 as poor agreement^76^.

## Results

The performance of the RF and RF_rfe models was assessed based on both the kappa values from the internal cross-validation, and the independent validation samples (prediction accuracy of the independent sample set and kappa values). The data pruning was carried out based on the SAGA wetness index, since this parameter had been identified as contributing most to the classification accuracy.

### Assessment based on the internal cross-validation

The kappa values varied with the different combinations of dataset and covariates with substantial agreement (ϰ between 0.4 and 0.8) between predicted and observed reference soil groups (Table 3). For both oversampled and non-oversampled training set, kappa values were lower when using the entire data (AllPT) compared to the record from the pruned dataset. The kappa values increased with increasing pruning level, either with the RF or the RF_rfe models but not consistently with the RF_rfe models based on the oversampled training set. Considering the pruned dataset, the RF_rfe models recorded the highest kappa values, though comparable records were observed for the non-oversampled and oversampled training set (Table 3).

**Table 3:** Percentage of Plinthosols samples removed from the total set, Training set (n), and kappa values of the different subsets of oversampled and non-oversampled data for models with (RF_rfe) and without (RF) recursive feature elimination.

	Data treatment	PT removed (%)	Non-oversampled training data			Oversampled training data		
			n	Kappa		N	Kappa	
RF	AllPT	—	792	0.48	(±0.12)	3096	0.53	(±0.08)
	90%CR	6.2	744	0.54	(±0.06)	2808	0.57	(±0.08)
	80%CR	12.4	696	0.56	(±0.07)	2520	0.59	(±0.09)
	SDCR	15.9	667	0.60	(±0.05)	2346	0.60	(±0.07)
RF_rfe	AllPT	—	792	0.52	(±0.08)	3096	0.55	(±0.08)
	90%CR	6.2	744	0.59	(±0.06)	2808	0.62	(±0.09)
	80%CR	12.4	696	0.61	(±0.06)	2520	0.59	(±0.08)
	SDCR	15.9	667	0.63	(±0.09)	2346	0.62	(±0.07)

PT: Plinthosols, AllPT: entire dataset, 90%CR: dataset with 5% lower and upper range pruning, 80%CR: dataset with 10% lower and upper range pruning, SDCR: dataset with standard deviation-based pruning.

### Assessment based on independent validation samples

The results of the RF and RF_rfe models for the oversampled and non-oversampled data including all the Plinthosols (AllPT) are presented in Table 4. It shows the confusion matrix between observed and predicted reference soil groups using the validation set. For all confusion matrices in the current study, each line represents the percentage of correct and incorrect classified samples and can be summed up to 100%. Correct predictions are located at the diagonal of the matrix. A high level of accuracy for the identification of the Plinthosols was observed with both oversampled and non-oversampled training set though the latter recorded the highest agreement between observed and predicted instances (91.5% for RF and 91.4% for RF_rfe). Irrespective of the models and the training set, most of the other reference soil groups were misclassified as Plinthosols. However, oversampling helped in this regard to reduce this misclassification for the Cambisols and Lixisols with the RF models on the one hand and for Cambisols, Gleysols, Leptosols with the RF_rfe models on the other hand (Table 4). Stagnosols were the most misclassified soil orders grouped to Plinthosols, except for the RF_rfe model based on the non-oversampled training set, which was able to accurately predict 33% of these soil groups.

**Table 4:** Confusion matrix between observed and predicted reference soil groups for the oversampled and non-oversampled data including all the Plinthosols with (RF_rfe) and without (RF) recursive feature elimination.

Observed	RF						Observed	RF_rfe					
	Predicted (%)							Predicted (%)					
	CM	GL	LP	LX	PT	ST		CM	GL	LP	LX	PT	ST
**Confusion matrix from models based on non-oversampled training data**													
CM	**5**.**9**	0	5.9	0	88.2	0	CM	**23**.**5**	5.9	0	5.9	64.7	0
GL	0	**64**.**3**	0	3.6	32.1	0	GL	0	**60**.**7**	0	0	39.3	0
LP	0	0	**75**	0	25	0	LP	0	0	**50**	0	50	0
LX	9.1	9.1	0	**18**.**2**	63.6	0	LX	0	9.1	0	**63**.**6**	27.3	0
PT	0.8	3.8	0.8	2.3	**91**.**5**	0.8	PT	1.6	7	0	0	**91**.**4**	0
ST	0	0	0	0	100	**0**	ST	0	0	0	0	66.7	**33**.**3**
**Confusion matrix from models based on oversampled training data**													
CM	**23**.**5**	0	11.8	0	64.7	0	CM	**47**.**1**	11.8	5.9	0	29.4	5.9
GL	0	**53**.**6**	0	10.7	32.1	3.6	GL	0	**64**.**3**	0	3.6	32.1	0
LP	25.0	0	**50**.**0**	0	25.0	0	LP	0	0	**75**.**0**	0	25.0	0
LX	9.1	9.1	0	**72**.**7**	9.1	0	LX	9.1	36.4	0	**27**.**3**	18.2	9.1
PT	8.5	5.4	1.6	3.1	**76**.**0**	5.4	PT	1.6	7.0	0	3.1	**86**.**8**	1.6
ST	16.7	0	0	0	83.3	**0**	ST	0	0	0	0	100.0	**0**

AllPT: entire dataset, CM: Cambisols, GL: Gleysols, LP: Leptosols, LX: Lixisols, PT: Plinthosols, ST: Stagnosols.

**Figure 4:**
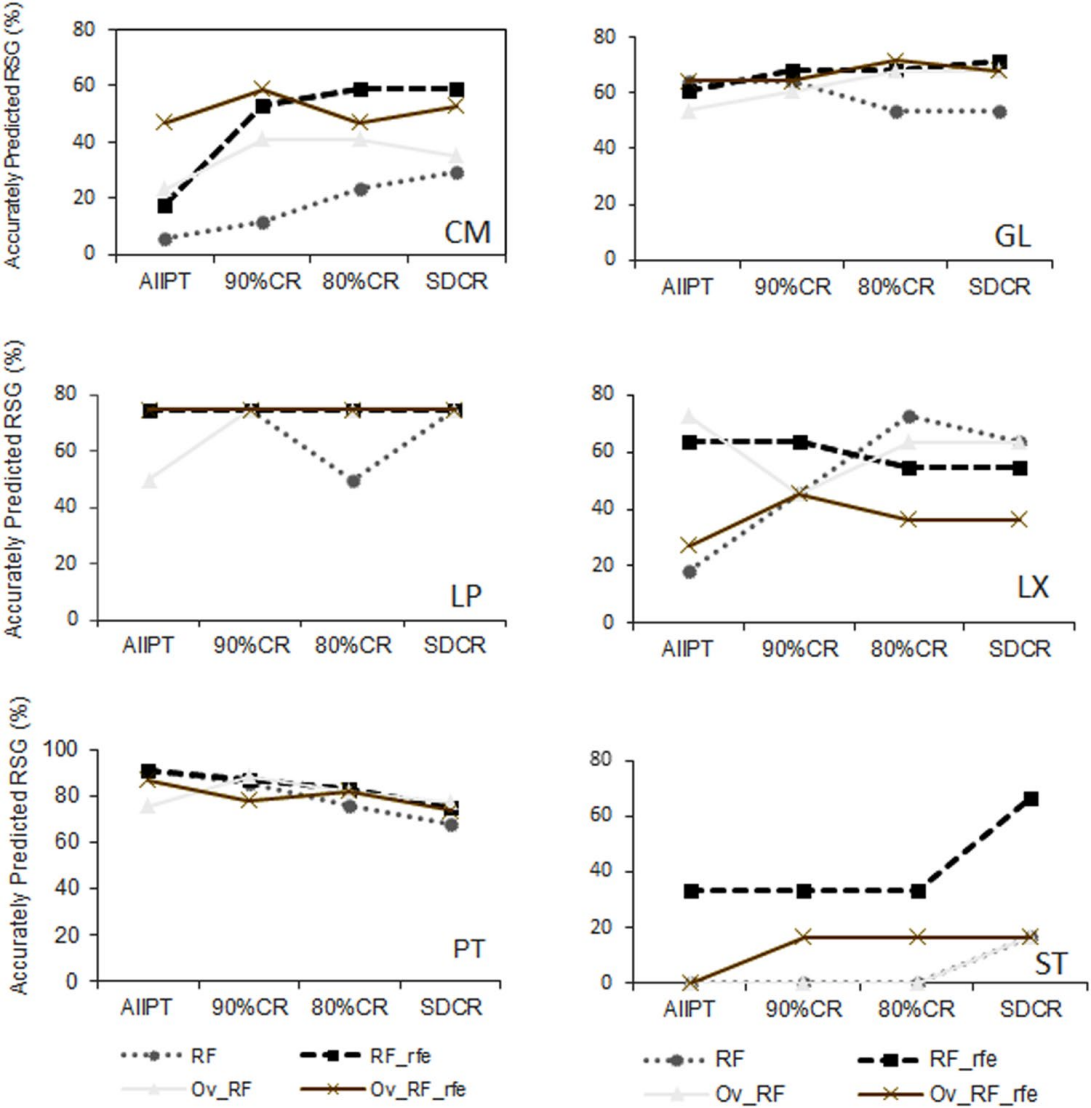
Accurately predicted reference soil groups by models with (RF_rfe) and without (RF) recursive feature elimination based on oversampled and non-oversampled datasets. CM: Cambisols, GL: Gleysols, LP: Leptosols, LX: Lixisols, PT: Plinthosols, ST: Stagnosols. AllPT: entire dataset including all Plinthosols, AllPT: entire dataset, 90%CR: dataset with 5% lower and upper range pruning, 80%CR: dataset with 10% lower and upper range pruning, SDCR: dataset with standard deviation-based pruning, Ov-RF: oversampling without recursive feature elimination, Ov_RF_rfe: oversampling with recursive feature elimination.

With increasing pruning level, gains in prediction accuracy were observed for most of the different reference soil groups (Fig. 4, see Supplementary Tables S3, S4). In most cases, the RF_rfe models performed better than the RF models especially with models based on non-oversampled training set. For instance, improvement in classification for the Cambisols was observed with the RF_rfe models when using the pruned datasets. Compared to the prediction based on the entire dataset (AllPT) for non-oversampled training set, these Cambisols gained approximately 35% in prediction accuracy with the 90%CR dataset and approximately 41% with both the 80%CR and SDCR datasets. For these same soil groups, the magnitude of the gain in prediction accuracy was much lower with the oversampled training set, with 6% and 12% respectively for the 90%CR and SDCR dataset.

With the RF_rfe models based on non-oversampled training dataset, the Gleysols recorded a 7% increase in prediction accuracy with both the 80%CR and 90%CR dataset, while the standard deviation core range (SDCR) improved by 10%. Again, the RF_rfe models based on random oversampling only improved accuracy by 3.6 to 7% for the Gleysols in the 80%CR and SDCR training datasets. With pruning, the RF_rfe model prediction for the Stagnosols resulted in a prediction accuracy of 33% and 16% for non-oversampled and oversampled dataset respectively. The highest increase (33%) in prediction accuracy for the Stagnosols was again recorded by the RF_rfe model based on non-oversampled SDCR training dataset. For Lixisols, prediction accuracy increased by 54 and 45% with the RF models using the non-oversampled 80% core range (80%CR) and standard deviation core range dataset (SDCR), respectively. Overall, pruning was thus effective in improving the prediction accuracy of the less abundant reference soil groups, and this positive effect was enhanced in the non-oversampled training sets compared to the oversampled ones, respectively.

No further improvement occurred for the Leptosols with data pruning. Compared to results from models based on the entire dataset (AllPT), the Plinthosol prediction accuracy dropped generally with increased pruning intensity, regardless of the models. RF_rfe models based on the oversampled 80%CR dataset and the non-oversampled 90%CR dataset recorded the lowest drop of 4.7% in prediction accuracy for the Plinthosols compared to the results with the entire dataset (AllPT).

**Figure 5:**
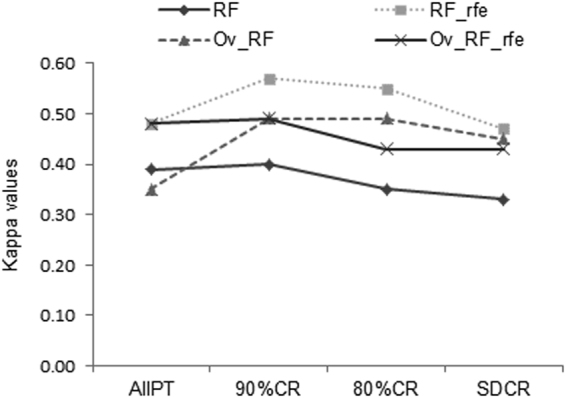
Variation of Kappa values for models with (RF_rfe) and without (RF) recursive feature elimination based on oversampled and non-oversampled datasets. AllPT: entire dataset including all Plinthosols, 90%CR: dataset with 5% lower and upper range pruning, 80%CR: dataset with 10% lower and upper range pruning, SDCR: dataset with standard deviation-based pruning, Ov-RF: oversampling without recursive feature elimination, Ov_RF_rfe: oversampling with recursive feature elimination.

From the models based on the original dataset (AllPT), the RF model based on the oversampled training set recorded the lowest kappa value. Kappa values (ϰ) generally increased with the 90%CR dataset and decreased with subsequent pruned datasets either with the RF or the RF_rfe models (Fig. 5). However, the RF_rfe models based on the non-oversampled training set recorded higher kappa values than the remaining models. Resulting models from the non-oversampled 90%CR and 80%CR dataset presented the highest kappa values, with ϰ = 0.57 and ϰ = 0.55, respectively.

### Variable importance

**Figure 6:**
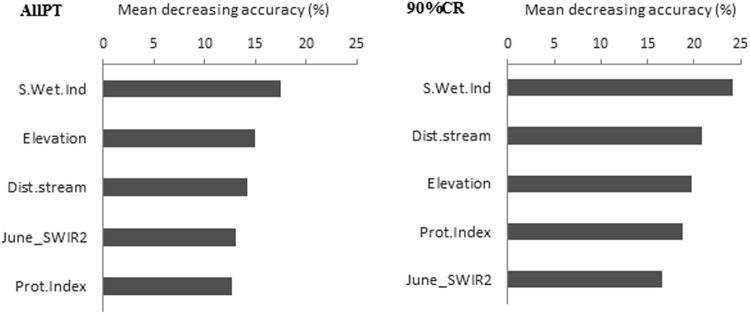
Variable importance for the recursive feature elimination models (non-oversampled AllPT and 90%CR). AllPT: entire dataset including all Plinthosols. 90%CR: dataset with 5% lower and upper range pruning, S.Wet.Index: Saga wetness index, Dist.stream: distance to streams, Prot.Index: protection index, June_SWIR: shortwave infrared acquired in June.

Although many models were considered in the present study with different datasets, results for the variable importance focused only on the RF_rfe models based on the non-oversampled entire dataset (AllPT) and the non-oversampled 90%CR dataset, since the latter presented the highest kappa value for the independent validation set. The others were omitted in order to avoid redundancy. Only the five top variables are presented in Fig. 6. For models based on the entire dataset (AllPT) and on the 90%CR dataset, the SAGA wetness index (S.Wet.Ind) was ranked as the most important covariate driving the reference soil group classification. For the 90%CR model, the wetness index was followed by the distance to stream network (Dist.stream), elevation, protection index (degree of local surface convexity or concavity) and the short-wave infrared acquired in June. Considering the different reference soil groups, the Gleysols discriminated significantly from the remaining groups by having the highest moisture level beside the Stagnosols and Lixisols, which also displayed relatively high moisture status (Table 5). However, the Gleysols differentiated from the latter and from other reference soil groups with the lowest distance to stream network and lowest position in the landscape.

**Table 5:** Kruskal–Wallis one-way analysis of variance of the main terrain parameters for the different reference soil groups based on the non-oversampled 90%CR dataset (90%CR).

RSG (n)	Wetness Index		Distance to stream (m)		Elevation (m)		Protection Index	
	mean	sd	mean	sd	mean	sd	mean	sd
Cambisols (n = 69)	7.82^a^	(±0.68)	647^a^	(±512)	313^a^	(±21)	0.03^a^	(±0.01)
Gleysols (n = 113)	8.72^b^	(±0.71)	242^b^	(±199)	287^b^	(±14)	0.02^b^	(±0.01)
Leptosols (n = 18)	6.03^c^	(±1.29)	857^c^	(±441)	372^c^	(±35)	0.06^a,c^	(±0.03)
Lixisols (n = 48)	8.26^d^	(±0.97)	569^a,d^	(±307)	293^b,d^	(±24)	0.02^b,d^	(±0.01)
Plinthosols (n = 467)	8.03^a,e^	(±0.4)	747^c,d,e^	(±515)	309^e^	(±20)	0.02^b,d,e^	(±0.01)
Stagnosols (n = 28)	8.46^b,d,f^	(±0.68)	947^c,f^	(±482)	309^a,e,f^	(±22)	0.02^b,d,e,f^	(±0.01)

RSG: reference soil group; letters (a. b, c, d, e, f) indicate whether the means are significantly different or not at p = 0.05. Same letters stand for no significant difference.

Stagnosols were characterized by the highest moisture level after the Gleysols, and by the highest distance to stream network with a lower protection index. The Lixisols revealed one of the highest moisture levels after the Stagnosols, in lower elevation and protection index areas as the Gleysols, but with a higher distance to stream. The moisture distribution, along with the distance to stream and elevation, also clearly differentiated between the Cambisols and the remaining reference soil groups; in particular, it singled out the Cambisols from the Leptosols, to which no significant difference was found regarding the protection index. The Leptosols were identified by their lowest soil moisture level as well as by their location at higher elevation and increased slope abundance (higher protection index), along with higher distance to the stream network. The Plinthosols discriminated from the remaining reference soil groups by their moisture distribution, along with the distance to stream for the Cambisols, Gleysols and Stagnosols while the elevation segregated them better from the Leptosols and Lixisols.

Figure 6 also shows that the terrain parameters took preeminence over the spectral data. The shortwave infrared (June_SWIR2) was listed only in fifth position after the terrain attributes for both the AllPT and 90%CR models. Overall, the contribution of the computed spectral indices was found to be negligible since they were not selected among the most important variables. The results further revealed that the spectral data acquired in June were the most prominent ones for the classification of reference soil groups in the Dano catchment.

### Spatial distribution of the reference soil groups

**Figure 7:**
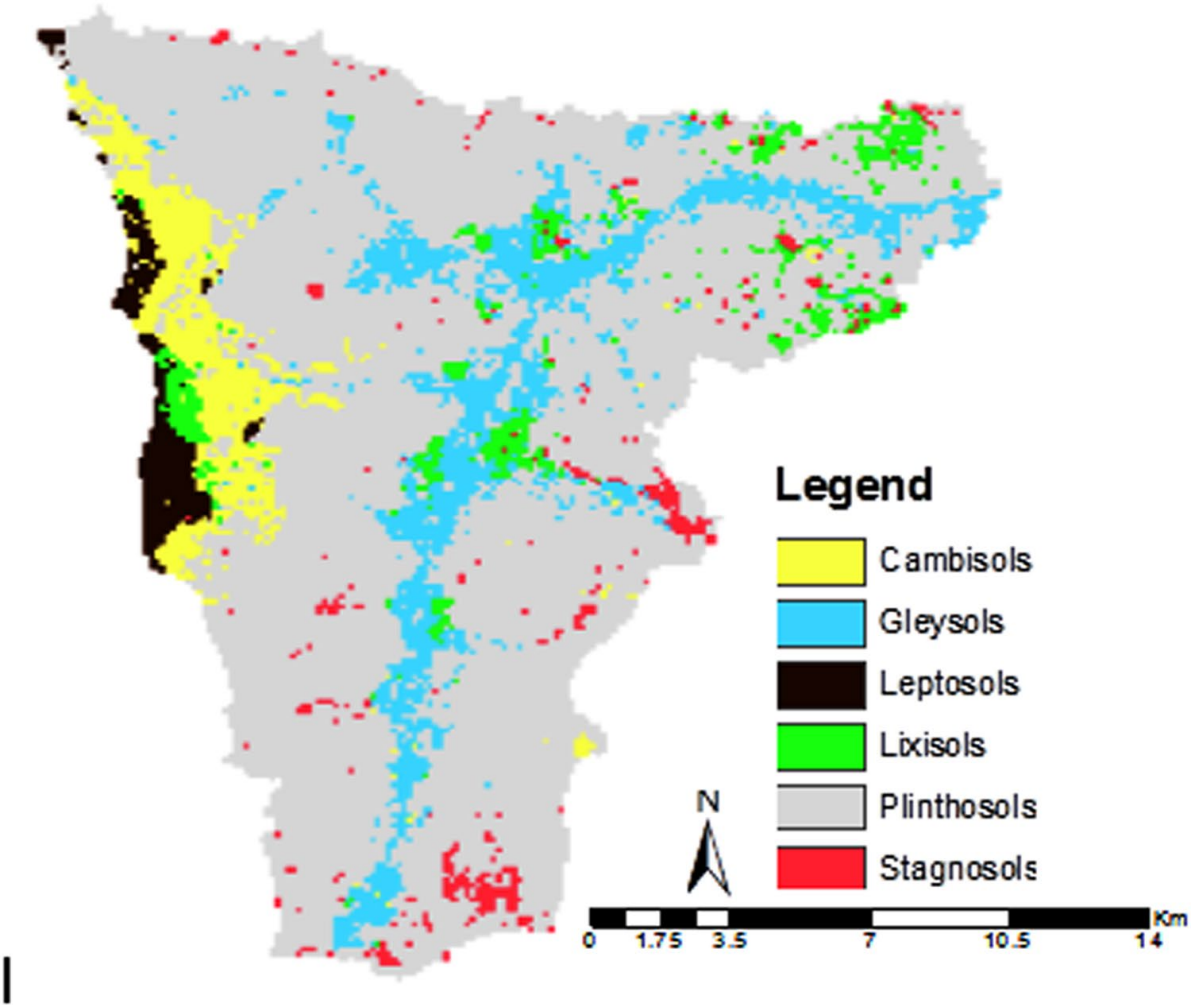
Spatial distribution of the reference soil groups for the recursive feature elimination model based on the dataset with 5% lower and upper range pruning (non-oversampled 90%CR) (the map was generated using ESRI ArcMap 10.3.1, www.esri.com).

Figure 7 shows the spatial distribution of the different reference soil groups as predicted by the RF_rfe model based on the non-oversampled 90%CR dataset, which provided the highest prediction accuracy for most of the smaller soil groups. The soils established on hard rock were classified as Leptosols by all models. Gleysols were predicted in the inland valleys, while soils predicted as Cambisols were generally located in the western part of the study area and mostly predicted in mid-slope regions. Lixisols were mapped in the lower elevation areas and spots of Stagnosols were displayed in various locations of the study area, especially in the southern and eastern parts. Plinthosols as the dominant soil group covered most of the landscape but were spatially restricted in the western area, where Leptosols and Cambisols were more abundant.

## Discussion

The RF and RF_rfe models that were based on the entire dataset (AllPT) resulted in relatively lower kappa values compared with other datasets with and without oversampling (Table 3). In addition, low prediction accuracies for the less abundant reference soil groups and an overestimation of the occurrence of Plinthosols were observed, especially with the non-oversampled training set (Table 4). This is mainly due to the skewed distribution of samples and the sharing of similar landscape and spectral properties within the covariate space. The latter dominated the misclassification error rate.

Oversampling did not prevent the overestimation of the Plinthosols with the independent validation set, although a lower magnitude of misclassification was recorded. Notably, models based on the oversampled training set yielded comparable kappa values at the modeling stage, especially with the pruned dataset. However, after recursive feature elimination, ROS provided lower kappa values compared with the RF_rfe models based on the non-oversampled training set at the validation stage. Consequently, oversampling the minority classes did not result in a significant improvement of the overall accuracies of the predictions. This observation is in line with the findings of Heung *et al*.^81^, who used ROS while predicting soil taxonomic units in Canada. They reported little to no improvement in prediction accuracy after oversampling.

Various studies beyond DSM have reported different and sometimes conflicting results over the performance of under- and oversampling in handling imbalanced dataset for classification^82–85^. This is mainly due to the fact that the performance of a particular resampling method is specific to the field of application and related datasets along with the learner involved. In our context, applying random oversampling to the original dataset (AllPT) recorded a lower kappa value (ϰ = 0.35) than other models dealing with the same dataset (Fig. 5). It is likely that duplication of instances by oversampling only increases the number of samples in a minority class, but does not actually generate new information about the class^82^, and overfitting can occur with poor performance when resulting models are applied to new dataset. This probably explains the underperformance of the oversampling approach when applied on the pruned dataset compared to the non-oversampled RF_rfe models, which provided better prediction with the validation set (Fig. 5).

When using the pruned dataset, the RF and RF_rfe models gained in prediction accuracy for most of the smaller reference soil groups such as the Cambislols, Gleysols, Stagnosols, and the Lixisols (Fig. 4, see Supplementary Tables S3 and S4). The improvement was observed for most reference soil groups, especially with models based on an objectively reduced set of covariates, which mostly performed better than the models using all covariates (Fig. 4). With recursive feature elimination, a higher prediction accuracy was observed for the less abundant reference soil groups while using the non-oversampled 90%CR and 80%CR dataset. Models based on these datasets also recorded the highest kappa value on the independent dataset (Fig. 5) showing substantial agreement between predicted and observed reference soil groups. Consequently, removing all Plinthosol points lower than 5% (or 10%) and higher than 95% (or 90%) of the cumulative percentage of the most important covariate (wetness index) resulted in a higher prediction performance and more suitable map results.

The better performance of the RF_rfe model and related core range (90%CR, 80%CR) can be related both to the use of feature selection and instance selection. Feature and instance selection correspond to the two main branches used within statistical learning to deal with the presence of noisy samples in a dataset^83^. The discriminative ability of covariates to differentiate between classes is weakened with the existence of overlapping pattern in the covariate space; for example, similar landscape characteristics for some classes, as was the case between the Plinthosols and the smaller soil groups in the study area. As pointed out by Ali *et al*.^82^, the overlapping covariate space results in the loss of the inherent capacity of these covariates to find sharp decision boundaries between classes. The issue of class overlapping has direct effect on most classifiers, which allocate the overlapping area to the majority class while considering the minority class as noise^84^. With the non-oversampled training set of the original data (AllPT), prediction with the validation set improved from a kappa value of ϰ = 0.39 when using all the covariates to ϰ = 0. 48 with the reduced set of covariates. As many authors have pointed out, feature selection results in keeping features with the most discriminative power with potential positive impact on classification prediction^85–87^. However, a higher level of classification could only be reached when feature selection was coupled with instance selection as already reported for the RF_rfe model based on the non-oversampled 90%CR training set.

For instance selection in the non-oversampled 90%CR and 80%CR training datasets, the removed sample points were located in the low-frequency range of the wetness index distribution. Considering the frequency distribution of many predictors, Qi^88^ pointed out that samples from the modal range are more characteristic of a particular soil class than those belonging to the lowest frequencies, which are referred to as potential source of noise. As Schmidt *et al*.^21^ observed, such an approach is hardly applicable when dealing with many soil covariates since each predictor should be singled out in the analysis. However, focusing on the frequency distribution of the main driving predictor in the present study has proven to be satisfactory with the improvement in prediction accuracy observed with the pruned dataset in general and with the 90%CR and 80%CR datasets in particular. These datasets includes the modal range of the wetness index, with the outer ranges being cut off. In many studies, the classification of profiles to a specific soil class is decided based on how their various morphological, physical, or chemical properties match the modal values of that particular class^89–91^. These profiles are then considered as the representative profiles. In the case of this study, the 90% core range, including the modal values of the wetness index, can be considered as more characteristic of the Plinthosols than the instances belonging to the margins. These latter instances could be considered as potential sources of noise. Results from several classification studies have showed that the presence of noise in classes related to a training dataset reduced the predictive accuracy of a learner on independent data^84,92,93^. Moreover, feature selection in a noisy environment (AllPT) led to lower performance. The results from the model based on the non-oversampled 90%CR and 80%CR training datasets showed that instance selection coupled with feature selection can improve classification prediction. As pointed out by Wasikowski and Chen^85^ as well as by Longadge and Dongre^94^, instance selection alone might not be sufficient in handling class imbalance issues, and in the context of this study, also to solve both class imbalance and noise issues.

The prediction accuracy of the Leptosols was not affected by the pruning carried out based on the wetness index. It appeared therefore that their learning by the RF and RF_rfe models did not improve upon the removal of the samples of the Plinthosols located within the margins of the wetness index. This could be related to the fact that these particular soil groups appear in different landscape positions by their establishment on hard rocks and their location at the highest elevation level compared to the remaining soil groups. A decrease in kappa values was observed with most of the models related to the standard deviation core range dataset (Fig. 5). This seems to suggest the SDCR as the pruning limit for the particular dataset of the current study, while revealing pruning of 5–10% as the potential range for model improvement.

Improving model accuracy might require either increasing the number of soil pedon observations for the small classes^39^, or assessing additional features that ameliorate the discrimination between the different soil groups. In addition to address scale related dependencies different multi- or hyperscale terrain attributes could reveal a better model performance and are able to increase the understanding of soil formation^25,95,96^. However, the current work suggests that instance selection associated with an optimal set of covariates can reduce the overwhelming influence of dominant soil groups, thus better allowing the expression of soil classes that have lower occurrences. Though comparison of different machine learning techniques was not in the scope of this study, the assessment of additional data mining algorithms could be further explored along with the multi- or hyperscale terrain attributes.

The terrain attributes drove the classification of the reference soil groups in the Dano catchment (Fig. 6) compared to the spectral data. The spectral data, standing mainly as proxy for the influence of vegetation on soil formation, have minor influence in the distribution of the soil groups in the study area. The feature selection algorithms selected the SAGA wetness index (S.Wet.Ind), followed by the distance to stream network (Dist.stream), elevation and the protection index (degree of local surface convexity or concavity) among the most important terrain attributes. These results are in line with the findings of Dobos *et al*.^97^, who reported an ascendency of terrain attributes such as slope, curvature, and potential drainage density over spectral data in temperate climates. Similarly, elevation and slope are ranked first, followed by spectral data for predicting soil classes^98^. The preeminence of the SAGA wetness index as a soil development factor in the Dano catchment suggests that the humidity regime is a key discriminatory element among the soil groups. The distance to stream, elevation, and the protection index may be seen along these lines as additional key regulatory parameters for soil moisture and related spatial distribution of the different soil groups.

The short infrared (SWIR from the band 6 of Landsat data) spectral data were most prominent when acquired in June (Fig. 6) for the classification of reference soil groups in the Dano catchment. This particular period corresponded to the plowing time. At that time, crops were absent or at an early stage of development, allowing satellite sensors to directly measure soil reflectance. It has been reported that soil moisture highly affects the SWIR reflectance^99^. Since the SWIR relates to soil moisture content, as is also the case for the Saga Wetness Index, it is obvious that soil moisture controlled the distribution of the reference soil groups over the Dano catchment. We assume that terrain and spectral covariate are complementary and that predicting reference soil groups for digital soil mapping thus relies heavily on concurrent soil-landscape characterization.

Soil groups located at lower elevations and closer to streams, such as Gleysols and Lixisols (Fig. 7), had higher moisture content than those located at higher altitude and more distant from streams, such as Leptosols and Cambisols. Also Stagnosols were characterized by high moisture level and generally located in flat areas. Thus, it made sense that wetness index that was responsible for the distribution of these less abundant soils contributed to the overall prediction accuracy. Lixisols were mainly located in lower elevation areas, as also reported by Gray *et al*.^100^, but Lixisols are usually less wet than the other soil groups mentioned. Hence, next to wetness, distance to streams and other terrain attributes were important variables for an accurate soil map prediction.

Leptosols were found at higher elevation and at larger distance to stream areas. These soils were well predicted by most of the models, since they were established on hard rock on the Ioba mountain, which fits the description of the WRB^68^. The spatial distribution of these Leptosols was consistent with the finding of Debella-Gilo *et al*.^101^, who found these soils mainly on hills and at the rocky part of the landscape. The presence of the major part of Cambisols next to the Leptosols might be attributed to erosion and deposition cycles, which are key elements for their distribution in high-elevation areas^68^. Vasques *et al*.^102^ also found Cambisols in sloping areas, subject to a more dynamic water flow.

Plinthosols have been found nearly at every position, thus occupying a major part of the landscape. These soils herein developed in level-to-gently-sloping areas with changing groundwater level or stagnating surface water^68^. This corresponds to the feature of the study area characterized by a flat and undulating landscape with altitude ranging between 259 and 465 m asl and an average slope gradient of 3.6%^67^. Plinthosols are soils characterized by Fe oxide accumulation under hydromorphic conditions. The change in moisture content (wetting and drying) results in the reallocation of dissolved Fe, leading to the constitution of Fe poor and Fe rich zones in the soil^103^. In the rainy season, mobilization and translocation of Fe^2+^ ions occurs due to reducing conditions, while the dry season gives rise to the oxidation of Fe^2+^ and precipitation of Fe oxides. As a result, Plinthosols are mainly hydromorphic soils^104^, the formation of which is greatly affected by moisture regime, as also evidenced by the Saga wetness index being the most important variable for the classification of the reference soil groups in the Dano catchment.

## Conclusion

This study focused on reducing the negative influence of a predominant reference soil group – the Plinthosols – on the spatial prediction of more seldom reference soil groups in tropical environments, in this case the Dano catchmen. For this purpose, we removed the Plinthosol samples falling within (i) the margins of the cumulative percentage of the density distribution and (ii) those found within the standard deviation of the values of the most important wetness index. This method was compared to the random oversampling approach.

The reference soil groups were predicted using random forest with and without recursive feature elimination. When using the entire dataset, we obtained lower prediction accuracies for most of the reference soil groups predicted as Plinthosols. However, increasing pruning intensity resulted in higher accuracies (increased kappa) especially with models based on non-oversampled dataset in combination with recursive feature elimination.

The best prediction was achieved when removing without oversampling all Plinthosol points lower than 5% and higher than 95% of the cumulative percentage of the most important variable (wetness index) with an optimal covariate set resulting from the recursive feature elimination. This improved classification accuracy by 7–41% relative to the prediction based on the entire dataset as the pruned samples, potential source of noise and redundant information, were removed. For this tropical environment, the moisture distribution (SAGA wetness index) was identified as the main driving factor for the reference soil group classification in the Dano catchment.

With the ongoing GlobalSoilMap.net initiative in Africa, soil mapping is being carried out using legacy data originating from different campaigns and involving many surveyors with some of these datasets confronted to source error issues. As demonstrated in this study, pruning combined with feature selection can improve classification accuracy. Therefore, this approach could be chosen as a particularly suitable alternative when in-field adjustments or post-processing reclassifications are not a viable option for creating soil maps.

## Electronic supplementary material

Supplementary information
