# Supplementary material for: Predicting reference soil groups using legacy data: A data pruning and Random Forest approach for tropical environment (Dano catchment, Burkina Faso)
Source: Sci Rep. 2018 Jul 2;8:9959. doi: 10.1038/s41598-018-28244-w (PMC6028482; doi:10.1038/s41598-018-28244-w)
Supplement: Supplementary file 1 — Supplementary information [file 41598_2018_28244_MOESM1_ESM.pdf]

Predicting reference soil groups using legacy data: a data pruning and Random Forest approach for tropical environment (Dano catchment, Burkina Faso)

Kpade O. L. Hounkpatin*[a], Karsten Schmidt[b], Felix Stumpf[c], Gerald Forkuor[d], Thorsten Behrens[b], Thomas Scholten[b], Wulf Amelung[a], Gerhard Welp[a]

[a]University of Bonn, Institute of Crop Science and Resource Conservation (INRES), Soil Science and Soil Ecology, Nussallee 13, D-53115 Bonn, Germany

[b]University of Tübingen, Department of Geosciences, Soil Science and Geomorphology, D-72070 Tübingen, Germany

[c]Agroscope - Swiss Federal Research Institute for Sustainability Soil Monitoring Network (NABO) - Modelling Unit, Reckenholzstrasse 191, CH - 8046 Zürich, Switzerland

[d] West African Science Service Centre on Climate Change and Adapted Land Use—WASCAL, Ouagadougou 06 P.O. Box 9507, Burkina Faso

*Corresponding author. hozias@uni-bonn.de

Supplementary Table S1. Count (n) and frequencies (%) of the reference soil groups in the Dano catchment

Reference soil groups	n	Percentage of grand total (%)
Cambisols (CM)	86	6.68
Gleysols (GL)	141	10.95
Leptosols (LP)	22	1.71
Lixisols (LX)	59	4.58
Plinthosols (PT)	645	73.45
Stagnosols (ST)	34	2.64

Supplementary Table S2. The distribution of the number of samples in different reference soil groups for each training set with and without oversampling. CM: Cambisols, GL: Gleysols, LP: Leptosols, LX: Lixisols, PT: Plinthosols, ST: Stagnosols. AllPT: entire dataset, 90%CR: dataset with 5 % lower and upper range pruning, 80%CR: dataset with 10 % lower and upper range pruning, SDCR: dataset with standard deviation-based pruning.

	Training set without oversampling						
Dataset	CB	GL	LP	LX	PT	ST	Total
AllPT	69	113	18	48	516	28	792
90%CR	69	113	18	48	468	28	744
80%CR	69	113	18	48	420	28	696
SDCR	69	113	18	48	391	28	667
	Training set with oversampling						
Dataset	CB	GL	LP	LX	PT	ST	Total
AllPT	516	516	516	516	516	516	3096
90%CR	468	468	468	468	468	468	2808
80%CR	420	420	420	420	420	420	2520
SDCR	391	391	391	391	391	391	2346

Supplementary Table S3. Confusion matrix between observed and predicted reference soil groups for the pruned dataset without oversampling and with (RF_rfe) and without (RF) recursive feature elimination. CM: Cambisols, GL: Gleysols, LP: Leptosols, LX: Lixisols, PT: Plinthosols, ST: Stagnosols; 90%CR: dataset with 5% lower and upper range pruning, 80%CR: dataset with 10% lower and upper range pruning, SDCR: dataset with standard deviation based pruning.

RF

	Predicted (%) : 90%CR					
Observed	CM	GL	LP	LX	PT	ST
CM	**11.8**	5.9	11.8	0	70.6	0
GL	0	**64.3**	0	0	32.1	3.6
LP	0	0	**75**	0	25	0
LX	9.1	18.2	0	**45.5**	27.3	0
PT	2.3	6.2	1.6	2.3	**85.3**	2.3
ST	0	0	0	0	100	**0**

	Predicted (%): 80%CR					
Observed	CM	GL	LP	LX	PT	ST
CM	**23.5**	0	11.8	0	64.7	0
GL	0	**53.6**	0	10.7	32.1	3.6
LP	25	0	**50**	0	25	0
LX	9.1	9.1	0	**72.7**	9.1	0
PT	8.5	5.4	1.6	3.1	**76**	5.4
ST	16.7	0	0	0	83.3	**0**

	Predicted (%) : SDCR					
Observed	CM	GL	LP	LX	PT	ST
CM	**29.4**	0	11.8	0	52.9	5.9
GL	3.6	**53.6**	0	10.7	32.1	0
LP	0	0	**75**	0	25	0
LX	9.1	9.1	0	**63.6**	9.1	9.1
PT	13.2	5.4	1.6	3.9	**68.2**	7.8
ST	16.7	0	0	0	66.7	**16.7**

RF_rfe

	Predicted (%) : 90%CR					
Observed	CM	GL	LP	LX	PT	ST
CM	**52.9**	5.9	0	5.9	35.3	0
GL	0	**67.8**	0	3.6	28.6	0
LP	0	0	**75**	0	25	0
LX	0	9.1	0	**63.6**	27.3	0
PT	3.1	7	0	1.5	**86.8**	1.6
ST	0	0	0	0	66.7	**33.3**

	Predicted (%) : 80%CR					
Observed	CM	GL	LP	LX	PT	ST
CM	**58.8**	5.9	0	5.9	29.4	0
GL	0	**67.9**	0	3.5	28.6	0
LP	0	0	**75**	0	25	0
LX	0	9.1	0	**54.5**	27.3	9.1
PT	3.8	7.8	0	1.6	**82.9**	3.9
ST	0	0	0	0	66.7	**33.3**

	Predicted (%) : SDCR					
Observed	CM	GL	LP	LX	PT	ST
CM	**58.8**	5.9	0	5.9	29.4	0
GL	0	**71.4**	0	3.6	25	0
LP	25	0	**25**	25	25	0
LX	0	0	0	**54.5**	27.3	18.2
PT	7	10.8	0	2.3	**75.2**	4.7
ST	0	0	0	0	33.3	**66.7**

Supplementary Table S4. Confusion matrix between observed and predicted reference soil groups for the pruned dataset under oversampling with (RF_rfe) and without (RF) recursive feature elimination. CM: Cambisols, GL: Gleysols, LP: Leptosols, LX: Lixisols, PT: Plinthosols, ST: Stagnosols; 90%CR: dataset with 5% lower and upper range pruning, 80%CR: dataset with 10% lower and upper range pruning, SDCR: dataset with standard deviation based pruning.

RF

	Predicted (%) : 90%CR					
Observed	CM	GL	LP	LX	PT	ST
CM	**41.2**	5.9	5.9	0.0	41.2	5.9
GL	0.0	**60.7**	0.0	3.6	32.1	3.6
LP	0.0	0.0	**75.0**	0.0	25.0	0.0
LX	9.1	18.2	0.0	**45.5**	27.3	0.0
PT	0.8	3.9	3.1	2.3	**88.4**	1.6
ST	0.0	16.7	0.0	0.0	83.3	**0.0**

	Predicted (%) : 80%CR					
Observed	CM	GL	LP	LX	PT	ST
CM	**41.2**	5.9	11.8	0.0	35.3	5.9
GL	3.6	**67.9**	0.0	3.6	21.4	3.6
LP	0.0	0.0	**75.0**	0.0	25.0	0.0
LX	9.1	18.2	0.0	**63.6**	9.1	0.0
PT	2.3	6.2	3.1	3.9	**82.2**	2.3
ST	0.0	0.0	0.0	0.0	100.0	**0.0**

	Predicted (%) : SDCR					
Observed	CM	GL	LP	LX	PT	ST
CM	**35.3**	5.9	11.8	0.0	41.2	5.9
GL	3.6	**67.9**	0.0	3.6	21.4	3.6
LP	0.0	0.0	**75.0**	0.0	25.0	0.0
LX	9.1	18.2	0.0	**63.6**	9.1	0.0
PT	3.9	5.4	2.3	2.3	**77.5**	8.5
ST	0.0	0.0	0.0	0.0	83.3	**16.7**

RFE

	Predicted (%) : 90%CR					
Observed	CM	GL	LP	LX	PT	ST
CM	**58.8**	5.9	5.9	11.8	17.6	0.0
GL	0.0	**64.3**	0.0	3.6	32.1	0.0
LP	0.0	0.0	**75.0**	0.0	25.0	0.0
LX	9.1	27.3	0.0	**45.5**	9.1	9.1
PT	4.7	7.8	0.0	4.7	**78.3**	4.7
ST	16.7	0.0	0.0	0.0	66.7	**16.7**

	Predicted (%) : 80%CR					
Observed	CM	GL	LP	LX	PT	ST
CM	**47.1**	11.8	5.9	0.0	29.4	5.9
GL	0.0	**71.4**	0.0	3.6	25.0	0.0
LP	0.0	0.0	**75.0**	0.0	25.0	0.0
LX	9.1	36.4	0.0	**36.4**	9.1	9.1
PT	3.1	7.8	0.0	3.9	**82.2**	3.1
ST	0.0	0.0	0.0	0.0	83.3	**16.7**

	Predicted (%) : SDCR					
Observed	CM	GL	LP	LX	PT	ST
CM	**52.9**	11.8	11.8	0.0	17.6	5.9
GL	3.6	**67.9**	0.0	3.6	25.0	0.0
LP	0.0	0.0	**75.0**	0.0	25.0	0.0
LX	9.1	36.4	0.0	**36.4**	9.1	9.1
PT	5.4	7.8	0.0	5.4	**73.6**	7.8
ST	0.0	0.0	0.0	0.0	83.3	**16.7**
